# Managing the Uncertainties Inherent in Prohibitive Voice: How Leadership Interacts With Employee Political Skill

**DOI:** 10.3389/fpsyg.2021.702964

**Published:** 2021-12-16

**Authors:** Xiaoxuan Wang, Fan Zhou

**Affiliations:** School of Management, Zhejiang University, Hangzhou, China

**Keywords:** authoritarian leadership, humble leadership, political skill, prohibitive voice, uncertainty management

## Abstract

Drawing from the uncertainty management theory, we examine how authoritarian leadership and humble leadership interact with employee political skill to predict prohibitive voice. We conducted a two-wave survey study of 43 managers and 176 subordinates in a power company in China. Our findings indicate that authoritarian leadership has a minimal negative effect on the psychological safety of employees with higher political skill, which in turn leads to a minimal negative effect on their prohibitive voice. Moreover, humble leadership is positively associated with prohibitive voice for employees with lower political skill. For employees with higher political skill, no type of leadership behavior has a significant influence on their prohibitive voice. We outline the implications of these findings for both theoretical and managerial practices.

## Introduction

Organizations are becoming increasingly reliant on employee voice, the expression of constructive opinions or concerns about work-related issues (Van Dyne and LePine, 1998), to ward off failures and achieve a continuous improvement (Detert and Burris, 2007). Meanwhile, employees’ opinions may contain promotive (e.g., new ideas intended to improve the *status quo*) or prohibitive (e.g., opinions about existing problematic practices at work) aspects (Liang et al., 2012). Compared to promotive voice, employees are often reluctant to express prohibitive voice because it does not always bring them desirable outcomes. Though some voicers may receive positive outcomes, such as higher performance evaluations (Thomas et al., 2010; Ng and Feldman, 2012; Burris et al., 2013), managers’ recognition and rewards (Burris, 2012), or more promotion opportunities (Dutton and Ashford, 1993), sometimes they may receive dislike or negative performance evaluations from managers when expressing explicit or implicit criticisms to *status quo* (De Dreu and Weingart, 2003; Bendersky and Hays, 2012; Burris, 2012). Employees’ worries stem from the uncertainties of personal benefits and costs (Takeuchi et al., 2012), which would inhibit their tendency to speak up about their concerns or criticisms. Considering the irreplaceable function of prohibitive voice, it is critical for organizations to help employees in effectively managing the uncertainties they may experience when deciding whether to use that voice.

Leaders usually have a strong influence in determining whether prohibitive voice is welcomed. Regarding correcting problematic practices and avoiding organizational failures, some leaders rely on subordinates’ ideas and suggestions, whereas others believe in their own authority and require subordinates’ unquestionable obedience. From this point of view, authoritarian leadership and humble leadership are such two types of behaviors contrasting with each other. Humble leadership values others’ ideas (Owens and Hekman, 2012), supports a workplace culture in which employees can readily admit mistakes at work, and focuses on improving work practices (Owens and Hekman, 2016). When working with such leaders, employees’ prohibitive voice would likely be viewed more favorably. In contrast, authoritarian leadership is typically efficiency oriented (Huang et al., 2015), usually emphasizes obedience, discipline, and unity (Farh and Cheng, 2000; Pellegrini and Scandura, 2008), and imposes severe punishments toward mistakes at work. According to the uncertainty management theory (UMT) (Van den Bos and Lind, 2002), employees usually search for social cues from interactions with their leaders to reduce their feelings of uncertainty. Leader’s expressed humility manifests appreciation to employee ideas, which signals that speaking up about concerns is relatively safe. Oppositely, leader’s authority may imply devaluing subordinates’ ideas and could be less likely to address employees’ perceived uncertainty. Briefly, the two types of leadership may differentially predict employees’ frequencies of expressing prohibitive voice, and examining these two types of leadership simultaneously may add new insights to the literature on prohibitive voice.

Meanwhile, the effectiveness of leadership and employees’ uncertainty management at work may be contingent on their individual differences (e.g., Griffin et al., 2007; Hochwarter et al., 2014). Psychological uncertainty derives from the inability to predict the future (Van den Bos and Lind, 2002), on the other hand, employees could initiate coping when faced with the stressed environment (Carver et al., 1989). Political skill, referring to an interpersonal capacity to effectively adjust behaviors according to different situations (Ferris et al., 2005), can serve as a capacity to cope with the uncertainties at work. On one hand, individuals with a high level of political skill can adjust their behaviors according to different situations and social partners. On the other hand, they look sincere and authentic to gain others’ trust, and thereby carrying out communications smoothly without conflicts (Ahearn et al., 2004; Ferris et al., 2005). This capacity would endow them with a sense of control and self-confidence (Kacmar et al., 2013), which makes their social environment at work more predictable (Kuhn and Sniezek, 1996; Van den Bos and Lind, 2002). On one hand, employees with a high level of political skill may be less susceptible to the negative effects of leaders’ destructive behaviors because they know how to navigate through uncertain work situations on their own rather than solely rely on resources from leaders. On the other hand, they may take better advantage of leaders’ constructive behaviors as they are adept at using social networks and opportunities in working contexts (Pfeffer, 1992; Ferris et al., 2005). We suggest that employee political skill is critical to address the uncertainties that are inherent in prohibitive voice, and it would be intriguing to explore how subordinates use their political skill in the interactions with leadership.

To address these important questions, our study draws on the UMT (Van den Bos and Lind, 2002) to first specify how authoritarian and humble leadership behaviors differentially predict employees’ prohibitive voice and also to highlight the role of employee political skill and the leader–follower interactions in the process of expressing prohibitive voice.

Our study makes several new contributions to the literature on voice. First, we draw on the UMT to explore the link between leadership and prohibitive voice and to compare the two distinct types of leadership behaviors (i.e., authoritarian leadership and humble leadership), which differentiate in the strategies of preventing mistakes and attitudes toward subordinates’ ideas. As engaging in prohibitive voice accompanies higher uncertainties and risks (Liang et al., 2012), it is valuable to investigate which kind of leadership can help employees to overcome apprehension about speaking up. We shed light on the different social cues stemming from authoritarian and humble leadership, and thereafter could contribute to an enhanced understanding of prohibitive voice through which a certain type of leadership behaviors is proved to be more effective than others. Such an effort answers the call for more research on the role of leadership in shaping employees’ voice behavior [see the meta-analysis by Chamberlin et al. (2017)].

Second, we adopt an actor–context interactionist perspective and examine employee political skill as an important boundary condition of leadership effectiveness in predicting prohibitive voice. Previous studies have tended to concentrate on how employees are passively affected by specific leadership behaviors (e.g., Detert and Burris, 2007; Walumbwa and Schaubroeck, 2009), however, followers are not always passive, helpless, or even victimized in the leader–follower dyad, and conversely, they could adopt their own capacity to cope with the uncertainties and insecurities that are inherent in an environment. To bridge this gap, researchers have called for more studies on the role of individual differences of followers in the leadership process (Antonakis et al., 2012). Accordingly, we explore the antecedents of prohibitive voice from the dyadic perspectives of supervisors and subordinates, and we propose that employee political skill could interact with leadership behavior in the prohibitive voice process. Additionally, the investigation of such a trainable characteristic may add a perspective emphasizing employees’ agency, suggesting that they can shape their work environment and take control of their own behaviors such as prohibitive voice.

## Development of Hypothesis

### Authoritarian Leadership, Political Skill, and Prohibitive Voice

Employees’ prohibitive voice is critical to reveal existing harmful practices, incidents, or behaviors at workplace. Because prohibitive voice is not always interpreted as positive, and sometimes its use may even incur interpersonal risks to voicers (Liang et al., 2012), employees may be willing to express prohibitive voice only when the inherent uncertainty about consequences is well managed.

The uncertainty management theory (Van den Bos and Lind, 2002) posits that psychological uncertainty derives from one’s inability to predict the outcomes in a given situation. Uncertainty about whether the leader would be open minded to subordinates’ challenging ideas could stimulate an avoidance tendency in regard to prohibitive voice (Liang et al., 2012; Takeuchi et al., 2012). According to this theory, employees search for information cues from leaders (Van den Bos et al., 1998; Van den Bos and Lind, 2002) to assess the uncertainties related to consequences of prohibitive voice and their leaders’ trustworthiness and also to judge whether it is safe to engage in such behaviors. On the other side, prior studies have found a significant variance in individuals’ ability to manage uncertainty (e.g., Griffin et al., 2007; Hochwarter et al., 2014). Therefore, we suggest an actor–context interactionist view—namely, that the effectiveness of managing the uncertainties that are inherent in prohibitive voice will be contingent on the interaction of employees’ individual differences and leadership behaviors.

In line with the UMT, we argue that authoritarian leaders will increase employees’ perceived uncertainty about the consequences of prohibitive voice. Authoritarian leaders are highly devoted to organizational efficiency (Huang et al., 2015), and they may be less likely to acknowledge employees’ ideas as their voice might potentially threaten the leader’s authority and diminish decision-making efficiency (Liang et al., 2012). Such an attitude toward subordinates’ ideas may dampen their willingness to speak up (Farh and Cheng, 2000). In addition, authoritarian leadership may trigger employees’ emotional fear (Wu et al., 2002) and increase their perceptions of the disadvantages associated with the uncertainties that are inherent in prohibitive voice. Consequently, even if employees have good intentions to help their organization, they will reconsider whether it is wise to express prohibitive voice, pondering whether they might be misunderstood by their leaders and subsequently encounter interpersonal and professional risks.

Nevertheless, we suggest that the impacts of authoritarian leadership will not be necessarily negative and, in fact, could be contingent on employees’ individual differences. Although several previous empirical studies have indicated that authoritarian leadership has a negative impact on voice (Li and Sun, 2015; Zhang et al., 2015), the meta-analytic results (Bedi, 2020) showed that the credibility interval for the correlation between authoritarian leadership and employee voice was wide and included zero, implying that moderators could account for the variability of this relationship (Whitener, 1990). For example, subordinates with higher authority orientation and dependence on leaders would likely to behave in a compliant manner when faced with authoritarian leadership, such as manifesting higher identification, more gratitude (Cheng et al., 2004), and greater loyalty *vis-à-vis* their leaders (Chou et al., 2005). We suspect that such favor seeking or loyalty might be a kind of political acting, which helps employees to gain trust from authoritarian leaders. We therefore propose that employees with higher political skill will be less susceptible to a negative influence of authoritarian leadership in their management of the uncertainties that are inherent in prohibitive voice.

Political skill is an important interpersonal capacity that enables one to effectively adjust one’s behaviors according to the specific situation, with the aim to influence others and achieve their personal and collective objectives (Ahearn et al., 2004). Specifically, highly politically skilled employees have a good sense of social astuteness and know how to appear sincere to gain others’ trust and support, so that they excel at dealing with different situations and social relationships (Ferris et al., 2005). Accordingly, we argue that political skill can serve as a capability to reduce psychological uncertainty as it provides employees with a sense of safety and self-confidence to control their environments and enhances their perception of behavioral predictability (Perrewé et al., 2005; Kacmar et al., 2013). Hence, politically skilled employees may feel more at ease when expressing their true thoughts in a more favorable and convincing way (Ferris et al., 2005) even if those ideas carry critical and challenging information. Thereafter, we suggest that their authoritarian leaders will pay more attention to the prosocial intentions and helpful content of the voice expressed by those employees with higher political skill, rather than focusing on the challenging nature of their behavior. In summary, employees with higher political skill will be less susceptible to the influence of authoritarian leadership and will be more likely to engage in prohibitive voice. In contrast, employees with lower political skill will rely more on their leaders to gain the resources needed to manage uncertainty and, therefore, will be less likely to enact prohibitive voice. Hence, we hypothesize:

*Hypothesis 1*: Perception of authoritarian leadership and political skill interactively influence prohibitive voice. Specifically, the relationship between authoritarian leadership and prohibitive voice is more negative for employees with lower political skill than for those with higher political skill.

### Psychological Safety as a Mediator

We also highlight the role of psychological safety, which has been widely regarded as a mediator between leadership antecedents and voice behavior (e.g., Detert and Edmondson, 2011; Liang et al., 2012). Psychological safety, defined as the extent to which employees believe their supervisors or coworkers will not punish nor misunderstand their risk-related behaviors (Detert and Burris, 2007), helps employees to reduce perceived workplace uncertainty (Schein and Bennis, 1965). Psychological safety has been proven to be positively associated with employee voice behavior, especially prohibitive voice (e.g., Ashford et al., 1998; Edmondson, 1999; Detert and Burris, 2007; Walumbwa and Schaubroeck, 2009; Liang et al., 2012). Leadership behavior is an influential factor in shaping employees’ perceptions of psychological safety (Edmondson, 2003), in which accessible, open-minded leaders can promote employees’ psychological safety. Conceivably, authoritarian leadership would lead to lower psychological safety and, in turn, inhibits employees’ tendency to express their ideas and concerns (Duan et al., 2018).

Our study investigates how employee political skill moderates the process in which authoritarian leadership impacts prohibitive voice through psychological safety. Specifically, higher political skill enables employees to effectively deal with different situations and to enhance the predictability of their work environment. When faced with authoritarian leadership, political skill can help to reduce the unease and anxiety, such that highly politically skilled employees can maintain a relatively high level of psychological safety and in turn will be more likely to express prohibitive voice. Conversely, employees with lower political skill are more susceptible to the negative impacts of authoritarian leadership on psychological safety and, therefore, will be less likely to express their concerns. Taken together, we propose a first-stage moderated-mediation hypothesis:

*Hypothesis 2:* Employees’ political skill moderates the indirect relationship between authoritarian leadership and prohibitive voice (*via* psychological safety), such that the indirect effect is more negative for employees lower in political skill.

### Humble Leadership, Political Skills, and Prohibitive Voice

Compared to authoritarian leadership that wards off mistakes at work by emphasizing obedience and centralized decision-making, humble leadership would admit their own limits and would welcome employees’ ideas to correct current problems. Humility was originally conceptualized as a personal trait or a virtue, which represents the highest level of self-awareness (Tangney, 2002). Owens et al. (2013) introduced this concept to I/O psychology studies and proposed the construct *humble leadership*, which could be characterized as (1) admitting their own limitations, (2) appreciating followers’ strengths and contributions, and (3) being teachable at work (Owens and Hekman, 2012). Unlike leaders with higher dominance who usually display higher resource holding potential and interactional control toward subordinates (Burgoon and Dunbar, 2006), humble leaders will manifest empathy, non-superiority, familiarity, and sincerity in the interactions with subordinates, for example, they may display a low tone of voice, small smiles, and slight head movements to show their modesty, and may also show their appreciation to the community *via* a verbal act of underestimating their own merits (D’Errico and Poggi, 2019). Such expressed humility also implies a willingness to be taught (Owens and Hekman, 2012), hence employees can easily get clear cues that their ideas will be favorably recognized.

Humble leaders provide employees with relatively high psychological capital (Walters and Diab, 2016), decrease their emotion exhaustion (Wang et al., 2018), and provoke their self-efficacy, optimism (Rego et al., 2017), trust, and positive effects (Liu, 2016). Accordingly, they help to reduce uncertainty by encouraging them to vocalize dissenting opinions and doubts (Owens and Hekman, 2012). Hence, employees who are under humble leaders feel more certain that they will not be criticized if they speak up with an idea that may challenge the *status quo*.

However, humble leadership may also lead to negative outcomes under some circumstances. As leaders’ expressed humility implicates a lack of dominance, less competent (Weidman et al., 2018), and hypocrisy (D’Errico, 2019), humble leaders may induce negative emotions such as sadness, bitterness, and anxiety, and in turn would be negatively evaluated by followers (Poggi and D’Errico, 2009; D’Errico, 2019), especially by those who have higher social dominance orientation (D’Errico, 2020). Such inconsistent findings implicate that the effectiveness of humble leadership could in part hinge on subordinates’ individual differences.

Accordingly, we speculate that political skill may affect the extent to which employees take advantage of humble leadership behaviors. Employees with higher political skill will benefit more when faced with humble leadership and making a decision about their own prohibitive voice behavior as they can more adeptly make use of opportunities and resources (Pfeffer, 1992) at work. For less politically skilled employees who lack a capacity to deal with uncertainty, leader’s expressed humility could also serve as a resource that helps them to cope with the uncertainties as humble leaders’ higher recognition of their value and greater openness to new ideas (Owens and Hekman, 2012; Owens et al., 2013) provide clear cues that voice is welcome and thereby dispel such employees’ discomfort. Nevertheless, as less politically skilled employees are less adept at taking advantage of opportunities at work, they may benefit less from humble leadership compared to their counterparts who are higher in political skill. Therefore, we hypothesize that:

*Hypothesis 3:* Perception of humble leadership and political skill interactively influence prohibitive voice. Specifically, the relationship between humble leadership and prohibitive voice is more positive for employees with higher political skill than for employees with lower political skill.

We also propose a first-stage moderated-mediation model in which political skill moderates the effect of humble leadership on prohibitive voice through psychological safety. As explained earlier, humble leadership will promote the use of employees’ prohibitive voice by enhancing employees’ psychological safety. Furthermore, employees with higher political skill will take better advantage of humble leadership to overcome the uncertainties and experience higher psychological safety in the process of using prohibitive voice. Likewise, less political skilled employees will feel safer in speaking up about their concerns when working with humble leaders though the effectiveness would be less salient as they are not as good as their counterparts with higher political skill at taking up such opportunities. Therefore, we hypothesize that:

*Hypothesis 4:* Employees’ political skill moderates the indirect relationship between humble leadership and prohibitive voice (*via* psychological safety), such that this indirect effect is more positive for employees higher in political skill.

**FIGURE 1:**
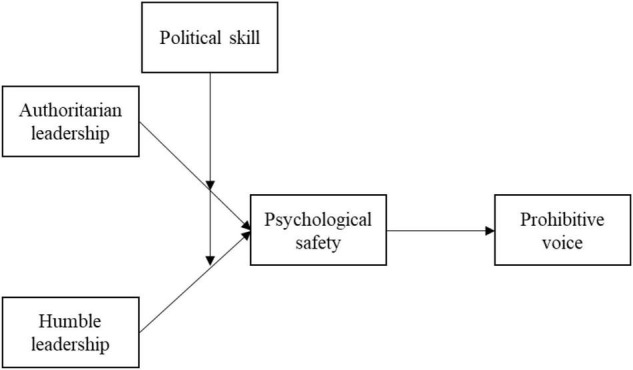
A hypothesized model.

Figure 1 illustrates our proposed model.

## Materials and Methods

### Sample and Procedures

We collected the data from managers and their subordinates employed in a large power company in China. As we aimed to study the antecedents of prohibitive voice, we needed a sample in which such behaviors are essential for organizational success and are easily observed. The power sector is a highly hazardous industry, in which employees need to stay alert at all times both to ensure grid reliability and to avoid accidents. Employee voice is crucial to eliminate hidden hazards in the workplace (Tucker et al., 2008) and ward off accidents and injuries (Barling et al., 2003). Moreover, the power enterprise is composed of a number of work units, in which members’ behaviors are affected by both their supervisors’ behaviors and their own dispositional factors. Thus, we used this sample after taking all of these factors into account.

All procedures were conducted in compliance with the American Psychological Association (APA) ethics code. To alleviate concerns about a common method variance, we collected the data from different sources (i.e., from supervisors and subordinates), through two rounds with a 1-month time lag (Podsakoff et al., 2003). We separated subordinates and supervisors by placing them in different rooms at the different survey times when answering questionnaires to avoid any psychological discomfort.

In the first wave (Time 1), we sent questionnaires to all 234 employees from 52 work units; we received 225 usable responses (the response rate of 96.2%). Respondents provided their perceptions of humble leadership, authoritarian leadership, and demographic information (age, gender, and tenure). In the second wave (Time 2), we collected the data from both subordinates and their immediate supervisors. The questionnaires were coded, ensuring that supervisors and subordinates could be matched. We received 176 employees’ responses from 43 work units (the response rate of 78.2%). Employees rated their political skill, psychological safety, and other control variables in the past month. In addition, we received 43 responses from supervisors, who rated their subordinates’ prohibitive voice in the past month. Thus, the final sample comprised 176 employees and their immediate supervisors.

On average, four employees reported to one supervisor. Within the sample, 52% were male, the average age was 35.34 years (SD = 9.91), and the average tenure was 13.60 years (SD = 11.69).

### Measures

We used translation/backtranslation procedures (Brislin, 1980) to ensure the equivalence of Chinese and English versions of items. Response options ranged from 1 (*strongly disagree*) to 5 (*strongly agree*) unless otherwise noted.

#### Subordinate Perceptions of Authoritarian Leadership

At Time 1, subordinates reported their immediate supervisor’s behaviors using the nine-item scale developed by Cheng et al. (2004), which was derived from a scale originally developed to assess paternalistic leadership. An example item is “My supervisor asks me to obey his/her instructions completely” (α = 0.87).

#### Subordinate Perceptions of Humble Leadership

At Time 1, subordinates rated humble leadership behavior of their supervisors with nine items from Owens et al.’s (2013) scale. Two example items are “My supervisor admits it when he/she does not know how to do something” and “My supervisor shows appreciation for the unique contributions of others” (α = 0.97).

#### Political Skill

At Time 2, we measured subordinates’ political skill using 14 items with the highest factor loadings from Ferris et al.’s (2005) scale. Items include “I spend a lot of time and effort at work networking with others,” “It is important that people believe I am sincere in what I say and do,” and “I am able to make most people feel comfortable and at ease around me.” These items were rated on seven-point scales (1 = strongly disagree to 7 = strongly agree) (α = 0.96).

#### Psychological Safety

At Time 2, we used six items to assess psychological safety from Liang et al.’s (2012) scale, which was based on Edmondson’s (1999) scale of group psychological safety. An example item is “Members of our units are able to freely express our thoughts.” Seven-point scales (1 = strongly disagree; 7 = strongly agree) were used to record responses (α = 0.95).

#### Prohibitive Voice

At Time 2, supervisors rated each immediate subordinate’s behaviors on a five-item scale developed by Liang et al. (2012). An example item is “During the past month, this person spoke up honestly with problems that might cause serious loss to our units” (α = 0.94).

#### Control Variables

We included subordinates’ age, gender, tenure, perceived status, and having ideas as control variables because of their potential impact on prohibitive voice. For instance, male employees may be more likely to speak up about their concerns; employees with a longer tenure may be more comfortable expressing opinions about some tough issues (Stamper and Van Dyne, 2001); employees with a relatively high status could feel more obligated to use their voice (Fuller et al., 2006; Janssen and Gao, 2015); and some employees rarely speak up just because they have nothing to say—that is, no ideas (Rusbult et al., 1988; Korsgaard and Roberson, 1995; LePine and Van Dyne, 1998; Van Dyne and LePine, 1998; Turnley and Feldman, 1999).

Age was reported as part of the employees’ personal information, and tenure was measured as the number of years worked in the work unit. We created dummy codes for gender (1 = male and 0 = female). We also measured *having ideas* (three items: “I have ideas about how to avoid serious loss to my unit,” “I have ideas about which factors can cause low efficiency in my unit,” and “I have ideas about how my job could be done better”; α = 0.89).

### Analysis Strategy

We used Mplus 7.0 (Muthén and Muthén, 1998) to examine our hypotheses. As we primarily investigate the dyadic supervisor–subordinate interactions, we conceptualized all variables in our model at an individual level although all participants were nested within their respective work units in our sample (i.e., 43 work units’ supervisors rated 176 subordinates’ prohibitive voice). To deal with the cluster sampling issue, we adopted a design-based modeling approach suggested by Wu and Kwok (2012), using the command “TYPE = COMPLEX” in Mplus, which enabled us to obtain estimates while taking the non-independence of observations into account with one single-level model. This approach is appropriate for our model as we theoretically focus on employees’ individual perception of leaders’ behaviors and their dyadic interactions, but do not expect differences between the individual and group level.

## Results

The descriptive statistics for the focal variables are shown in Table 1.

**TABLE 1:** Correlations, means, SDs, and reliabilities.

Variables	*M* (level 1)	SD (level1)	*M* (level 2)	SD (level 2)	1	2	3	4	5	6	7	8
(1) Gender	0.55	0.50	0.54	0.37	1	0.24	0.31*	–0.05	0.19	0.15	0.36*	0.19
(2) Tenure	13.60	11.69	13.58	7.31	0.15	1	0.31*	0.05	0.10	0.14	0.19	–0.02
(3) Have idea	3.56	0.77	3.59	0.55	0.19*	0.06	1	0.06	0.19	0.29	0.13	–0.07
(4) Authoritarian leadership	2.85	0.77	2.83	0.56	0.08	0.16*	0.06	1	−0.54**	0.38*	–0.01	–0.19
(5) Humble leadership	4.02	0.77	4.00	0.47	0.02	0.01	0.19*	−0.20**	1	–0.14	0.18	0.13
(6) Political skill	5.02	1.08	4.98	0.98	0.17*	0.09	0.17*	0.26**	0.03	1	0.37*	0.16
(7) Psychological safety	5.61	0.95	5.61	0.73	0.24*	0.12	0.07	0.01	0.12	0.42**	1	0.46**
(8) Prohibitive voice	3.79	0.77	3.77	0.65	0.24*	–0.05	0.00	–0.06	0.07	0.18*	0.37**	1

*The lower diagonal reports the results of the correlations at the individual level of analysis (level 1); for the group variables, the group value is assigned to each individual. The higher diagonal reports the results of the correlations at the group level of analysis (level 2); the individual variables are aggregated to calculate the group mean. Mean and SD in the first column refer to the individual-level variables, while mean and SD in the second column refer to the group-level variables. Sample size: Level 1 = 163–176 due to missing data; Level 2 = 43. Employee gender dummy coded 0 = female, 1 = male.*

*p < 0.05; **p < 0.01.

To examine interactive effects, all predictors were grand-mean centered to avoid the multicollinearity issue (Aiken et al., 1991). We controlled for employees’ gender, tenure, perceived status, and having ideas in the tests. All results reported are standardized estimates.

**FIGURE 2:**
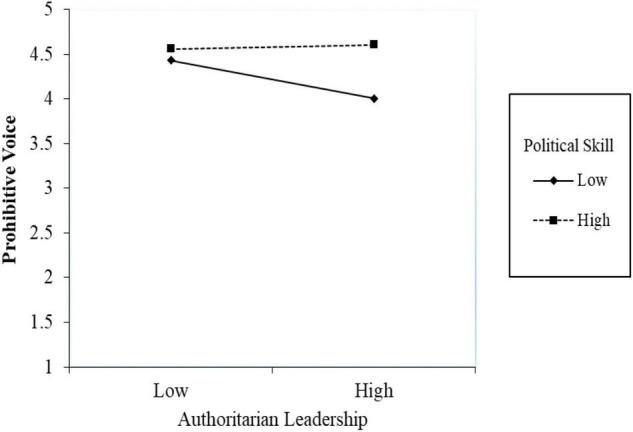
Simple slope for the interaction effect of authoritarian leadership and political skill on prohibitive voice.

First, to examine Hypothesis 1, we regressed prohibitive voice onto authoritarian leadership, political skill, and the interaction term. As shown in Table 2, in Model 1, the interaction of authoritarian leadership and political skill was not significant (β = 0.18, SE = 0.12, *p* = 0.12). Thus, Hypothesis 1 is not supported, but still the interactive pattern is plotted in Figure 2.

**TABLE 2:** The results of the regression analysis of leadership behavior, political skill, and prohibitive voice.

Variable	Model 1: Prohibitive voice	Model 2: Prohibitive voice	Model 3: Psychological safety	Model 4: Psychological safety	Model 5: Prohibitive voice	Model 6: Prohibitive voice
Intercept	5.22	5.24	6.18	6.18	3.54	3.52
**Controls**						
Gender	0.24**	0.22**	0.19*	0.17*	0.19*	0.17*
Tenure	–0.10	–0.12	0.03	0.02	–0.10	−0.12^†^
Having ideas	−0.12*	−0.11*	–0.11	–0.09	–0.09	−0.09^†^
**Independent variables**						
Authoritarian leadership (AL)	−0.12^†^		–0.07		–0.10	
Humble leadership (HL)		0.08		0.13		0.05
**Moderator**						
Political skill (PS)	0.24**	0.23*	0.53**	0.50**	0.10	0.09
AL × PS	0.18		0.35**		0.09	
HL × PS		−0.21*		−0.29*		–0.13
**Mediator**						
Psychological safety (PS)					0.27**	0.28**
Residual variance	0.85	0.86	0.65	0.69	0.81	0.81
	**Δ indirect effect**				**95% CI**	
AL × PS → PS → Prohibitive voice	0.16				CI = (0.032, 0.286)	
HL × PS → PS → Prohibitive voice	–0.21				CI = (–0.420, 0.000)	

*No of observations = 157; No of participants = 176. Standardized estimates.*

^†^p < 0.10; *p < 0.05; **p < 0.01.

**FIGURE 3:**
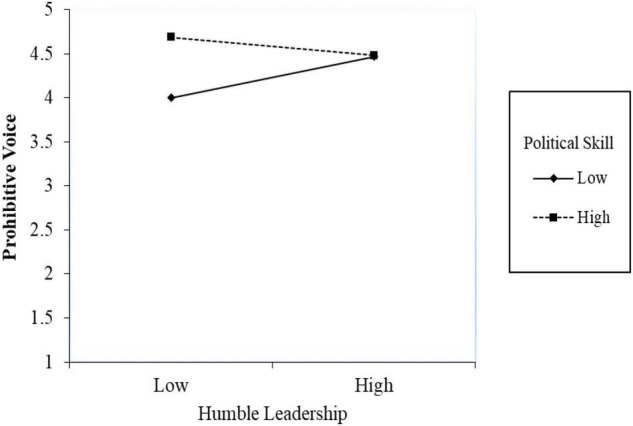
Simple slope for the interaction effect of humble leadership and political skill on prohibitive voice.

We used a similar approach to regress prohibitive voice onto humble leadership, political skill, and the interaction term. Model 2 in Table 2 reveals that the interactive effect of the perception of humble leadership and political skill on prohibitive voice is significant (β = –0.21, SE = 0.11, *p* = 0.04). The simple slope tests (Aiken et al., 1991) showed that humble leadership had a significant positive relationship with prohibitive voice for those employees with lower political skill (β = 0.30, SE = 0.15, *p* = 0.04). However, as opposed to our hypothesis, the effect of humble leadership was not significant for employees with higher political skill (β = –0.14, SE = 0.10, *p* = 0.17). Figure 3 plots the interactive effects based on our data. Thus, Hypothesis 3 about the interactive effect of humble leadership and political skill was found to be significant, but the pattern was the opposite of our expectation.

**FIGURE 4:**
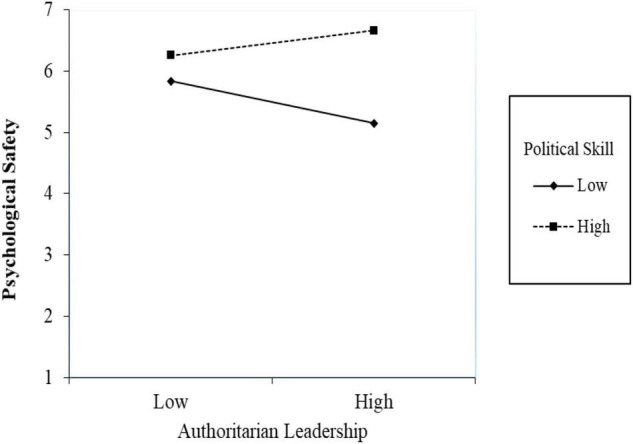
Simple slope for the interaction effect of authoritarian leadership and political skill on psychological safety.

Our study posits a first-stage moderated-mediation model (Edwards and Lambert, 2007), in which employees’ political skill moderates the indirect relationship between leadership behavior and prohibitive voice. We first examined the moderating effect of political skill on the relationship between leadership and prohibitive voice. As indicated by Model 3 in Table 2, the interaction of authoritarian leadership and political skill is positively related to psychological safety (β = 0.35, *p* < 0.01). Figure 4 shows that, among employees with lower political skill, authoritarian leadership has a significant negative impact on psychological safety (simple slope β = –0.44, *p* < 0.01). In contrast, for respondents with higher political skill, authoritarian leadership was positively related to psychological safety (simple slope β = 0.26, *p* < 0.05). We next calculated the proposed conditional indirect effect and examined its significance. The results showed that the indirect effect of authoritarian leadership on prohibitive voice through psychological safety was significant among employees with higher political skill [indirect effect = 0.06, 95% CI = (0.00, 0.12)], whereas it was not significant among those with lower political skill [indirect effect = –0.10, 95% CI = (–0.20, 0.00)]. The difference between these two conditional indirect effects was significant [Δ indirect effect = 0.16, 95% CI = (0.03, 0.29)]. These findings support Hypothesis 2.

**FIGURE 5:**
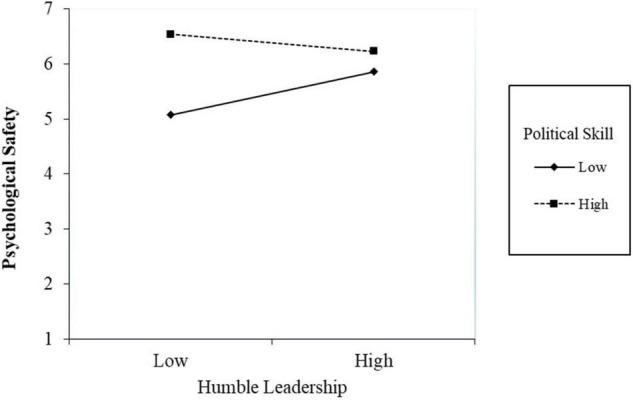
Simple slope for the interaction effect of humble leadership and political skill on psychological safety.

Following a similar rationale, we examined the moderated-mediation model in which political skill moderates the indirect effect of humble leadership on prohibitive voice *via* psychological safety. As shown in Model 4 in Table 2, the interaction of humble leadership and political skill is negatively associated with psychological safety (β = –0.29, *p* < 0.05). Figure 5 shows that for employees with lower political skill, humble leadership has a significant positive impact on psychological safety (simple slope β = 0.51, *p* < 0.05). In contrast, among employees with higher political skill, humble leadership had a slight negative impact on psychological safety though it was not significant (simple slope β = –0.20, *p* = 0.12). However, the conditional indirect effects of humble leadership on prohibitive voice *via* psychological safety were not significant at all levels of political skill [–1 SD: indirect effect = 0.14, 95% CI = (–0.01, 0.30); + 1 SD: indirect effect = –0.07, 95% CI = (–0.15, 0.01)]. The difference between the conditional indirect effects was not significant either [Δ indirect effect = –0.21, 95% CI = (–0.42, 0.00)]. Thus, Hypothesis 4 was not supported.

## Discussion

This study examined how leadership behaviors (i.e., authoritarian leadership and humble leadership) interact with employees’ individual differences (i.e., political skill) to predict prohibitive voice. We found that authoritarian leadership was negatively related to prohibitive voice among employees with lower political skill, whereas humble leadership was positively related to prohibitive voice for such employees; in contrast, employees with higher political skill were likely to engage in more prohibitive voice behavior compared to their counterparts, regardless of how their leaders behaved. In addition, political skill buffered a negative impact of authoritarian leadership on psychological safety, such that employees with higher political skill maintained a relatively high level of psychological safety, and in turn were more willing to express their prohibitive voice.

The pattern of interaction between humble leadership and political skill was contrary to our expectations. Nevertheless, when all of the results were taken together, we found the person–supervisor fit to be an important factor in motivating employee prohibitive voice. Specifically, employees who are lower in political skill may benefit more from humble leadership but may be victimized more by authoritarian leadership. In contrast, employees who are more politically skilled may be less susceptible to either humble leadership or authoritarian leadership. Hence, our study has several implications for future research.

### The Joint Effect of Leadership and Employee Political Skill on Prohibitive Voice

Our findings identified a significant joint effect of leadership behavior and employees’ individual differences in predicting prohibitive voice, which is consistent with actor–context interactionist perspectives (e.g., Woodman and Schoenfeldt, 1990). In the past, the literature on voice has documented that leaders’ behavior is a critical factor in motivating employee voice, and researchers have diligently sought to determine the ideal type of leadership behavior (e.g., Detert and Burris, 2007; Detert and Treviño, 2010; Liu et al., 2010; Zhang et al., 2015). Extending the current literature, our study compares the two contrasting types of leadership—authoritarian leadership and humble leadership—and reveals that neither of them have an overall significant negative or positive direct effect on prohibitive voice, yet both can influence certain employees. The interaction of authoritarian leadership and political skill was not significant in our study though this outcome may reflect the limited sample size. However, the simple slope tests indicated that authoritarian leadership significantly hinders those less politically skilled employees’ prohibitive voice, whereas employees with higher political skill are not significantly affected by the leadership type. These results partly address the call in a prior meta-analysis (Bedi, 2020) to determine moderators of the relationship between authoritarian leadership and voice. In brief, although Hypothesis 1 about the interactive effect of authoritarian leadership and political skill was not supported, the moderating role of employee political skill in the leadership–voice relationship remains an intriguing concept to investigate, and it could be viewed as an antidote to some putatively unfavorable leadership behaviors, such as authoritarian leadership.

In support of Hypothesis 3, our findings indicated that humble leadership interacts with employee political skill to influence prohibitive voice though the pattern we identified is the opposite of our expectation. These findings suggest that employees with lower political skill would be better off with humble leaders, compared with their counterparts who are higher in political skill, possibly because they can get more support from leaders to cope with the uncertainties stemming from the unpredictable nature of the outcomes of their behaviors (Owens and Hekman, 2012). As a result, these employees are likely to engage in more prohibitive voice as opposed to their counterparts who work with less humble leaders, as shown in the simple slope tests. As for relatively political skilled employees, we found that humble leadership was not significantly related to their prohibitive voice behavior—contrary to our hypothesis that they would take better advantage of humble leadership and would speak up with their concerns more frequently. We speculate that such politically adept employees may already be capable of effectively dealing with the uncertainties that are inherent in prohibitive voice, so that they will not benefit from humble leaders to the same extent as those employees who are lower in political skill. Furthermore, as the leader’s image is generally linked to power and dominance, employees with a better sense of social astuteness could instinctively suspect if the leader’s humble behavior is a self-presentational strategy or even hypocrisy (Sezer et al., 2018), rather than sincere willingness to listen to subordinates’ voice. Thus, we speculate that highly political skilled employees tend to withhold their ideas to avoid the possible retaliation from “false humble” leaders. To sum up, such a result warrants further investigation in the future.

Overall, our results suggest that there is no certain type of leadership behavior that is necessarily always positive or negative; instead, both leadership behavior and individual differences matter for fostering prohibitive voice. Hence, our study provides a scope for considering the other factors that might moderate the leader–voice relationship and for investigating the complex interactive mechanism of individual and context.

### The Mediating Role of Psychological Safety

Our findings also demonstrated that political skill moderates the indirect effect of authoritarian leadership on prohibitive voice *via* psychological safety. Specifically, employees who are higher in political skill experience relatively high psychological safety when faced with authoritarian leadership, which also allows them to express more prohibitive voice. In contrast, authoritarian leadership negatively predicted psychological safety among employees with lower political skill in our study, but the conditional indirect effect was not significant. Such a result can also account for the lack of support for our Hypothesis 1, which deals with the direct effect of interaction of authoritarian leadership and political skill, by highlighting the mediating role of psychological safety. Psychological safety is influential in predicting prohibitive voice—a topic that has been examined in previous research (Liang et al., 2012)—because it helps to mitigate the uncertainties derived from the potential personal costs induced by prohibitive voice. We encourage future research on voice to pay more attention to the UMT and to further examine when and how employees speak up from an uncertainty management perspective.

Based on our data, the proposed moderated-mediation model in which political skill moderates the relationship between humble leadership and prohibitive voice through psychological safety was not significant. In turn, we speculate that the psychological safety may not be the most salient psychological mechanism to explain how humble leadership impacts prohibitive voice. Moreover, the UMT may be not sufficient for explaining the antecedents of prohibitive voice; thus, we discuss several alternative propositions later in this manuscript that may bolster our model. Additionally, our sample size was limited, which may partly explain the insignificant results. Further investigation is therefore needed to more deeply explore the interaction of humble leadership and employees’ political skill.

### Practical Implications

Our research findings concerning the antecedents of prohibitive voice have several implications for managerial practices. First, our findings indicate that employees with higher political skill will remain in a relatively high state of psychological safety no matter how their leaders behave, which helps them to manage uncertainty, so that thereafter they tend to express more prohibitive voice. In line with the substitute for leadership theory (Kerr and Jermier, 1978), we infer that political skill might act as a substitute for resources provided by leadership, helping employees to better manage environmental uncertainty and taking greater control over their own behaviors. The current literature indicates that employees can build and develop political skill through mentoring and work experiences (Ferris et al., 2005). Thus, it is important for organizations to consider how to foster such a capacity in both employees and managers, such as through mentoring, training programs, or other developmental exercises to help employees build political skill.

Second, our study findings highlight the role of person–supervisor fit in motivating prohibitive voice. Authoritarian leadership generally emphasizes employees’ unconditional obedience (Farh and Cheng, 2000) and is less open to their ideas—attitudes that evoke employees’ emotional fear (Wu et al., 2002) and team emotional exhaustion (Chiang et al., 2021). These side effects are likely to have an especially strong influence on less political skilled employees because they lack the capacity to address psychological uncertainty and unsafety at work. Such employees may benefit more from working with humble leaders, who have higher tolerance for their mistakes and give more recognition to employees’ inputs (Owens and Hekman, 2012), thereby helping employees to address the discomfort inherent in the prohibitive voice process. Thus, managers should tailor their strategies toward different employees. To facilitate this nuanced approach, organizations can provide supervisory mentoring programs to enhance person–supervisor fit (Payne and Huffman, 2005) and can design training programs for supervisors aimed at improving their skills when interacting with subordinates.

### Limitations and Future Directions

Despite the strengths of our research design, such as using multi-source and multi-wave data to reduce the common method bias, it also has several limitations.

First, the sample size was not large enough and was limited to employees within a single organization in a non-Western culture. The sample size problem may account for the unsupported hypotheses. In addition, limiting our investigation to just the members of one Chinese company may reduce the generalizability of our findings to other industries and cultures. For example, authoritarian leadership is generally linked to negative consequences such as lower employee satisfaction (Cheng et al., 2002) and lower organizational commitment (Farh et al., 2006). On the other hand, authoritarian leadership may be aligned with the aspects of traditional Chinese culture, which emphasize paternalistic hierarchy and high power distance, and it is fair for leaders to behave in an authoritarian way and demand absolute compliance and respect (Cheng et al., 2004; Liden, 2012). Thus, authoritarian leadership will not necessarily be viewed as negative by some Chinese employees if they strongly adhere to this traditional cultural notion. Besides, the effects of humble leadership may also vary from culture to culture. As Confucian values and collectivism, which permeate through Chinese culture (Ou et al., 2014), regard humility as a virtue or a positive leader characteristic (Fu et al., 2010; Koo and Park, 2018), humble leadership would likely bring positive outcomes in Chinese population. However, in some individualist culture, leaders’ humility may not be seen as a good characteristic and may not always lead to positive consequences. For example, humble politicians could be interpreted as submissive, less competent, or hypocritical and in turn trigger followers’ negative emotions and evaluations (Poggi and D’Errico, 2009; D’Errico, 2019). In addition, the effectiveness of humble leadership could largely hinge on follower’s individual characteristics, e.g., people with relatively high social dominance orientation would evaluate humble leaders more negatively, whereas people higher in self-esteem and moral sensitivity would favor humble leaders more (D’Errico, 2020). Accordingly, it is valuable for future research to investigate more cultural and individual boundary conditions for the effectiveness of humble and authoritarian leadership in other populations.

Second, we operationalized and measured leadership variables (i.e., authoritarian leadership and humble leadership) at the individual level. We deemed this approach to be appropriate because our research focused on the dyadic interactions between the leader and employee, and how individual employees react within that relationship. Nevertheless, this operationalization and measurement prevented us from examining the potential cross-level effects of aggregated group-level leadership on individual-level prohibitive voice behavior. Future research could use a multi-level design and a larger sample to investigate the person–supervisor interactions at the group level, as well as their cross-level effects on employee individual outcomes.

Third, our results for the moderated-mediation effects may be susceptible to the common method bias as there is no time lag between the assessment of the mediator (i.e., psychological safety) and the dependent variable (i.e., prohibitive voice). Fortunately, we used supervisor-rated prohibitive voice as the dependent variable, which somewhat mitigated the same-source quandary. Nevertheless, we call for time-lagged, longitudinal, or quasi-experimental designs to further examine the underlying mediating mechanism of prohibitive voice.

In addition, in our moderated-mediation model, the indirect effect of humble leadership on prohibitive voice *via* psychological safety was not significant—so we suggest future research to explore alternative theories to explain the effects of humble leadership. For example, resource-related theory, such as the job demand-resource model (Demerouti et al., 2001; Demerouti and Bakker, 2008), may help clarify how humble leadership fosters prohibitive voice. Notably, humble leadership provides employees with job-related psychological resources, such as social supports and freedom (Walters and Diab, 2016; Rego et al., 2017), which can activate the motivational process and enhance employees’ work engagement (Owens et al., 2015). In turn, highly engaged employees may be more willing to enact proactive behaviors (Blader and Tyler, 2009), such as prohibitive voice. We suggest future research to explore how work engagement transmits the interaction between humble leadership and political skill.

Additionally, we suggest that social learning perspective (Bandura, 1977) is used as an alternative to explain how humble leadership influences prohibitive voice. In line with the social learning theory, when leaders manifest relatively high humility, employees would emulate such behaviors, creating a collective sense of humility within the team—namely, all members would be willing to acknowledge mistakes and try to learn from others (Owens and Hekman, 2016). As a consequence, employees would likely express their concerns freely. Thus, it would be intriguing to explore how humble leadership fosters prohibitive voice by creating a learning climate.

## Conclusion

In summary, our research suggests that neither authoritarian leadership nor humble leadership has a direct effect on employee prohibitive voice, but the interaction of leadership and employee political skill matters in this process. Specifically, authoritarian leadership harms the psychological safety of employees with lower political skill and in turn stifles their prohibitive voice, but has a weaker impact on employees with higher political skill. Humble leadership motivates employees who are lower in political skill to engage in more prohibitive voice, but is less beneficial for employees who are higher in political skill. By highlighting the importance of person–supervisor fit in the process of prohibitive voice, our research offers new insights into how and why different leadership behaviors influence prohibitive voice.

## Data Availability Statement

The raw data supporting the conclusions of this article will be made available by the authors, without undue reservation.

## Ethics Statement

The studies involving human participants were reviewed and approved by Zhejiang University. The patients/participants provided their written informed consent to participate in this study.

## Author Contributions

XW performed the statistical analysis and wrote the first draft of the manuscript. FZ revised the draft critically for important intellectual content and contributed to revision of the manuscript. XW and FZ contributed to the conception and design of the study, read, and approved the submitted version.

## Conflict of Interest

The authors declare that the research was conducted in the absence of any commercial or financial relationships that could be construed as a potential conflict of interest.

## Publisher’s Note

All claims expressed in this article are solely those of the authors and do not necessarily represent those of their affiliated organizations, or those of the publisher, the editors and the reviewers. Any product that may be evaluated in this article, or claim that may be made by its manufacturer, is not guaranteed or endorsed by the publisher.
